# Correction: Thangameeran et al. Examining Transcriptomic Alterations in Rat Models of Intracerebral Hemorrhage and Severe Intracerebral Hemorrhage. *Biomolecules* 2024, *14*, 678

**DOI:** 10.3390/biom14081034

**Published:** 2024-08-20

**Authors:** Shaik Ismail Mohammed Thangameeran, Sheng-Tzung Tsai, Hock-Kean Liew, Cheng-Yoong Pang

**Affiliations:** 1 Institute of Medical Sciences, Tzu Chi University, Hualien 97004, Taiwan; 106324122@gms.tcu.edu.tw (S.I.M.T.); flydream.tsai@gmail.com (S.-T.T.); 2 Neuro-Medical Scientific Center, Hualien Tzu Chi Hospital, Buddhist Tzu Chi Medical Foundation, Hualien 97004, Taiwan; 3 Department of Neurosurgery, Hualien Tzu Chi Hospital, Buddhist Tzu Chi Medical Foundation, Hualien 97004, Taiwan; 4 PhD Program in Pharmacology and Toxicology, Tzu Chi University, Hualien 97004, Taiwan; 5 Department of Medical Research, Hualien Tzu Chi Hospital, Buddhist Tzu Chi Medical Foundation, Hualien 97004, Taiwan

In the original publication [1], there was a mistake in Figure 1A. In the corrected image, the labels of ICH and Severe ICH have been swapped to accurately reflect the conditions represented. The figure now correctly shows the ICH condition in the top row and the Severe ICH condition in the bottom row. We have corrected Figure 1A and the complete Figure 1 with the corrected Figure 1A appears below; the corresponding caption follows. The images of the brain slices used for the hematoma calculation are provided in Table S1. In response to the addition of Supplementary Materials (Table S1), a corresponding citation has been incorporated into Section 3.1 of the main text.

The authors state that the scientific conclusions are unaffected. This correction was approved by the Academic Editor. The original publication has also been updated.

## Figures and Tables

**Figure 1:**
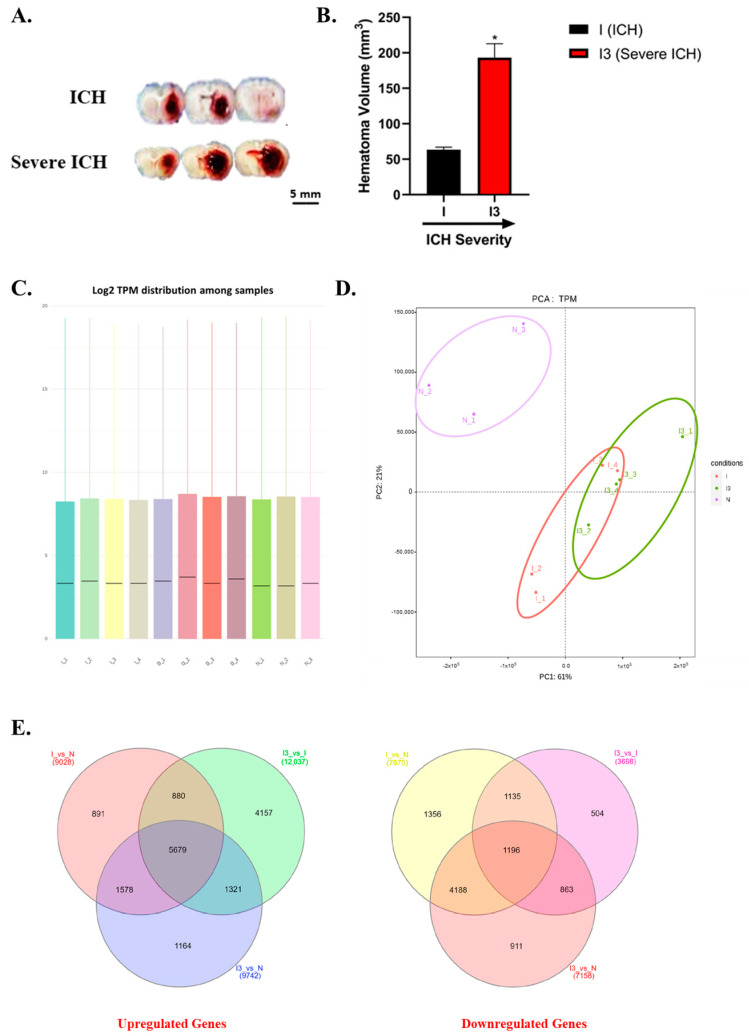
Comprehensive analysis of intracerebral hemorrhage severity and transcriptomic changes. Representative images of brain slices from ICH and severe ICH are shown (**A**). The volume of the striatal hematomas formed in the ICH (I) and severe ICH (I3) is quantitated (**B**); volume in mm^3^ for I and I3 (* *p* < 0.05). The hematoma in the severe ICH rats is nearly 3-fold larger than that in the ICH rats. Box plots illustrating the distribution of log2-transformed transcripts per million (TPM) (**C**). The Principal Component Analysis (PCA) scatter plot depicts the segregation of transcriptomic profiles between the brain tissue from normal (N), ICH (I), and severe ICH (I3) animals (**D**). The ellipses (purple: N, red: ICH, and green: severe ICH) represent a 95% confidence interval for the dispersion of the conditions in the multidimensional space. Venn diagrams (**E**) showing the overlap of differentially expressed genes between conditions: (**left**) upregulated genes in I versus N, I3 versus I, and I3 versus N, respectively; (**right**) downregulated genes in the same comparative groups (*n* = 3 for N and *n* = 4 for I and I3, respectively).

## Supplementary Materials

The following supporting information can be downloaded at: https://www.mdpi.com/article/10.3390/biom14081034/s1, Table S1: RNA-sample-list-for-RNA-sequence.
